# Prognostic value of the CALLY index for functional outcomes in acute ischemic stroke: a systematic review and exploratory quantitative synthesis

**DOI:** 10.3389/fneur.2026.1849476

**Published:** 2026-08-07

**Authors:** Zongyu Cai, Shihuan Zhong, Yifan Liang

**Affiliations:** 1 Yulin Traditional Chinese Medicine Hospital, Yulin, Guangxi, China; 2 School of Information and Management, Guangxi Medical University, Nanning, Guangxi, China

**Keywords:** acute ischemic stroke, albumin, CALLY index, C-reactive protein, lymphocyte, prognosis, systematic review

## Abstract

**Background:**

The C-reactive protein-albumin-lymphocyte (CALLY) index is a novel composite biomarker reflecting systemic inflammation, nutritional status, and immune function. Emerging evidence suggests its potential prognostic value in acute ischemic stroke (AIS). However, no systematic analysis has been conducted to evaluate its association with functional outcomes in AIS patients. This systematic review with exploratory quantitative synthesis aimed to assess the prognostic value of the CALLY index for functional outcomes in AIS.

**Methods:**

We systematically searched the Cochrane Library, EMBASE, PubMed, and Web of Science databases from inception to March 2026. The search was initially conducted in February 2025 and updated in March 2026 prior to submission. Observational studies evaluating the association between the CALLY index and functional outcomes in AIS patients were included. The primary outcome was poor functional outcome defined by the modified Rankin Scale (mRS), with poor outcome defined as mRS ≥ 2, mRS > 2, or mRS ≥ 3 at 3 months. Pooled odds ratios (ORs) with 95% confidence intervals (CIs) were calculated using a random-effects model. Study quality was assessed using the Newcastle-Ottawa Scale (NOS). Heterogeneity was evaluated using Cochran’s *Q* test and *I*^2^ statistic.

**Results:**

Three studies involving 1,161 AIS patients were included. The exploratory quantitative synthesis revealed that a higher CALLY index was significantly associated with a lower risk of poor functional outcome (pooled OR = 0.766, 95% CI: 0.695–0.845, *p* < 0.001), with no significant heterogeneity across studies (*I*^2^ = 0.0%, *Q* = 1.33, *p* = 0.516). All included studies were of high quality (NOS scores 8-9). Publication bias could not be reliably assessed due to the limited number of included studies.

**Conclusion:**

This systematic review with exploratory quantitative synthesis provides preliminary pooled evidence suggesting that the CALLY index may be a potential predictor of functional outcome in AIS patients. A higher CALLY index was associated with a lower risk of poor functional outcomes. However, given the limited number of included studies (only three retrospective studies from China), these findings should be considered hypothesis-generating rather than confirmatory and require validation in large-scale, multicenter, prospective studies across diverse populations before clinical application.

**Systematic review registration:**

The review was registered in PROSPERO: CRD420261350196 https://www.crd.york.ac.uk/prospero/display_record.php?ID=CRD420261350196.

## 1 Introduction

Acute ischemic stroke (AIS) remains a leading cause of mortality and disability worldwide, accounting for approximately 7.8 million new cases annually and imposing a substantial burden on patients, healthcare systems, and society (1, 2). China bears one of the highest stroke burdens globally, with approximately 17.8 million prevalent cases and 3.4 million new events reported in 2020 (3). Despite significant advances in acute treatment strategies, including intravenous thrombolysis and endovascular thrombectomy (EVT), a substantial proportion of patients continue to experience unfavorable functional outcomes (4, 5). Therefore, early identification of patients at higher risk for poor outcomes is essential for guiding targeted interventions, implementing personalized management strategies, and optimizing clinical care to improve long-term rehabilitation outcomes.

Several prognostic biomarkers reflecting individual pathophysiological dimensions have been investigated in AIS (6). The neutrophil-to-lymphocyte ratio (NLR) and the systemic immune-inflammation index (SII) capture aspects of inflammatory and immune responses, while the Prognostic Nutritional Index (PNI) and C-reactive protein-to-albumin ratio (CAR) reflect nutritional and inflammatory status (7–9). However, each of these markers captures only a single or limited aspect of the complex pathophysiological milieu, potentially limiting their predictive performance. Moreover, while the National Institutes of Health Stroke Scale (NIHSS) remains the standard for assessing stroke severity, laboratory-based biomarkers that can be rapidly obtained at admission may offer complementary prognostic information beyond clinical scores alone.

The C-reactive protein-albumin-lymphocyte (CALLY) index, calculated as albumin × lymphocyte count/(CRP × 10), is a novel composite biomarker that uniquely integrates three key pathophysiological dimensions: C-reactive protein (CRP) as a marker of systemic inflammation, albumin as a nutritional and anti-inflammatory indicator, and lymphocyte count as a parameter of immune competence (10–12). CRP, as an acute-phase reactant, reflects the intensity of the systemic inflammatory response and has been associated with larger infarct volumes and recurrent vascular events (13–15). Albumin possesses antioxidant properties, maintains endothelial integrity, and serves as a marker of nutritional reserve (16). Lymphocytes reflect adaptive immune function, and stroke-induced lymphopenia has been associated with post-stroke infection and poor prognosis (17, 18). By integrating these three dimensions into a single metric, the CALLY index may provide a more comprehensive assessment of a patient’s overall pathophysiological state than any individual biomarker.

Initially proposed for predicting prognosis in hepatocellular carcinoma, the CALLY index has shown promising prognostic value in several malignancies and has recently attracted attention in cardiovascular and cerebrovascular diseases (19–21). Its clinical accessibility, low cost, and comprehensive nature make it a particularly attractive candidate for prognostic assessment in stroke patients.

Recent studies have begun exploring the prognostic utility of the CALLY index in AIS, examining its association with hemorrhagic transformation, functional outcomes, stroke-associated pneumonia, and early neurological deterioration. While several studies have reported associations between lower CALLY index values and unfavorable outcomes, differences in study design, sample size, outcome definitions, and patient populations have yielded heterogeneous findings, limiting the generalizability of individual study results. To date, no systematic review has been performed to synthesize the available evidence on this topic.

Therefore, this study aimed to conduct a systematic review with exploratory quantitative synthesis to evaluate the association between the CALLY index and functional outcomes in patients with AIS, and to assess the overall quality and consistency of evidence supporting its prognostic value.

## 2 Methods

This systematic review with exploratory quantitative synthesis was conducted in accordance with the Preferred Reporting Items for Systematic Reviews and Meta-Analyses (PRISMA) 2020 guidelines (22). The protocol was registered in PROSPERO (registration number: [CRD420261350196]).

### 2.1 Search strategy

A comprehensive literature search was performed across four electronic databases: Cochrane Library, EMBASE, PubMed, and Web of Science, from database inception through February 2025. An updated search was conducted in March 2026 prior to manuscript submission; no additional eligible studies were identified. The search strategy combined Medical Subject Headings (MeSH) terms, EMTREE terms (for EMBASE), and free-text keywords related to three concept domains: (1) the CALLY index (“CALLY index” OR “CRP-albumin-lymphocyte” OR “C-reactive protein albumin lymphocyte” OR “CRP-ALB-LYM”); (2) ischemic stroke (“ischemic stroke” OR “cerebral infarction” OR “acute ischemic stroke” OR “brain ischemia” OR “brain infarction” OR “cerebral ischemia”); and (3) prognosis (“prognosis” OR “outcome” OR “functional outcome” OR “modified Rankin Scale” OR “disability” OR “mortality”). Boolean operators (AND/OR) were used to combine search terms across domains. No language restrictions were applied. Reference lists of relevant articles were manually screened to identify additional eligible studies. Grey literature sources, including ClinicalTrials.gov and preprint servers, were not systematically searched.

### 2.2 Inclusion and exclusion criteria

Studies were included if they met the following criteria: (1) original observational studies with longitudinal follow-up (prospective or retrospective cohort designs); (2) enrolled adult patients (≥18 years) diagnosed with AIS; (3) evaluated the CALLY index as a prognostic biomarker; (4) reported multivariable-adjusted odds ratios (ORs) with 95% confidence intervals (CIs) for the association between the CALLY index (per unit increase as a continuous variable) and functional outcomes; and (5) defined functional outcome using the modified Rankin Scale (mRS), with poor outcome defined as mRS ≥ 2, mRS > 2, or mRS ≥ 3 at 3 months. Studies were excluded if they: (1) were reviews, case reports, editorials, or conference abstracts without full data; (2) enrolled patients with non-ischemic stroke or other neurological diseases; (3) used a modified CALLY formula; (4) reported only categorical (e.g., high vs. low) but not continuous effect estimates.

The variation in mRS thresholds across included studies was acknowledged *a priori*. All definitions represent clinically meaningful thresholds of functional disability, and the impact of this variation was assessed through heterogeneity testing and sensitivity analysis.

### 2.3 Study selection and data extraction

Two reviewers independently screened titles and abstracts of all identified records, followed by full-text assessment of potentially eligible studies. Disagreements were resolved through discussion or consultation with a third reviewer. Data were extracted using a standardized form including: first author, publication year, study design, country, sample size, patient characteristics (age, sex distribution), treatment modality, CALLY index calculation method, outcome definitions, adjusted OR values with 95% CIs, and covariates included in the multivariable analysis.

### 2.4 Quality assessment

The methodological quality of included studies was assessed using the Newcastle-Ottawa Scale (NOS) for observational studies (23, 24). The NOS evaluates three domains: selection (4 items, maximum 4 stars), comparability (1 item, maximum 2 stars), and outcome assessment (3 items, maximum 3 stars), yielding a total score ranging from 0 to 9 stars. Studies scoring ≥7 were considered high quality, 4–6 moderate quality, and <4 low quality. Two reviewers independently assessed each study, with discrepancies resolved by consensus. It should be noted that high NOS scores reflect methodological rigor in study design and reporting but do not eliminate the inherent limitations of retrospective observational studies, including potential residual confounding and selection bias.

### 2.5 Statistical analysis

The primary outcome measure was the pooled OR with 95% CI for the association between the CALLY index (per unit increase as a continuous variable) and poor functional outcome. All three included studies reported adjusted ORs per unit increase in the CALLY index from multivariable logistic regression models. Log-transformed ORs and their standard errors were calculated from the reported ORs and 95% CIs using the formula: SE = [ln (upper CI) – ln (lower CI)]/(2 × 1.96). We acknowledge that with only three studies, the DerSimonian-Laird estimator may underestimate between-study variance, and alternative methods (e.g., Hartung-Knapp adjustment, restricted maximum likelihood) may provide more robust confidence intervals. However, given the negligible observed heterogeneity, the choice of estimator is unlikely to materially alter the results. Leave-one-out sensitivity analysis was performed by sequentially excluding each study and recalculating the pooled effect estimate to assess the robustness of findings and the influence of individual studies. Heterogeneity across studies was assessed using Cochran’s Q test (significance threshold *p* < 0.10) and quantified by the *I*^2^ statistic. An *I*^2^ value of 0–25% indicated negligible heterogeneity, 25–50% low heterogeneity, 50–75% moderate heterogeneity, and >75% high heterogeneity. A random-effects model (DerSimonian-Laird method) was used to pool the estimates regardless of heterogeneity level, as it provides more conservative estimates and accounts for between-study variability. Given the limited number of studies (*n* = 3) publication bias was not assessed. All statistical analyses were performed using R software (version 4.2.3, R Foundation for Statistical Computing, Vienna, Austria). A two-sided *p*-value <0.05 was considered statistically significant.

## 3 Results

### 3.1 Study selection

**Figure 1:**
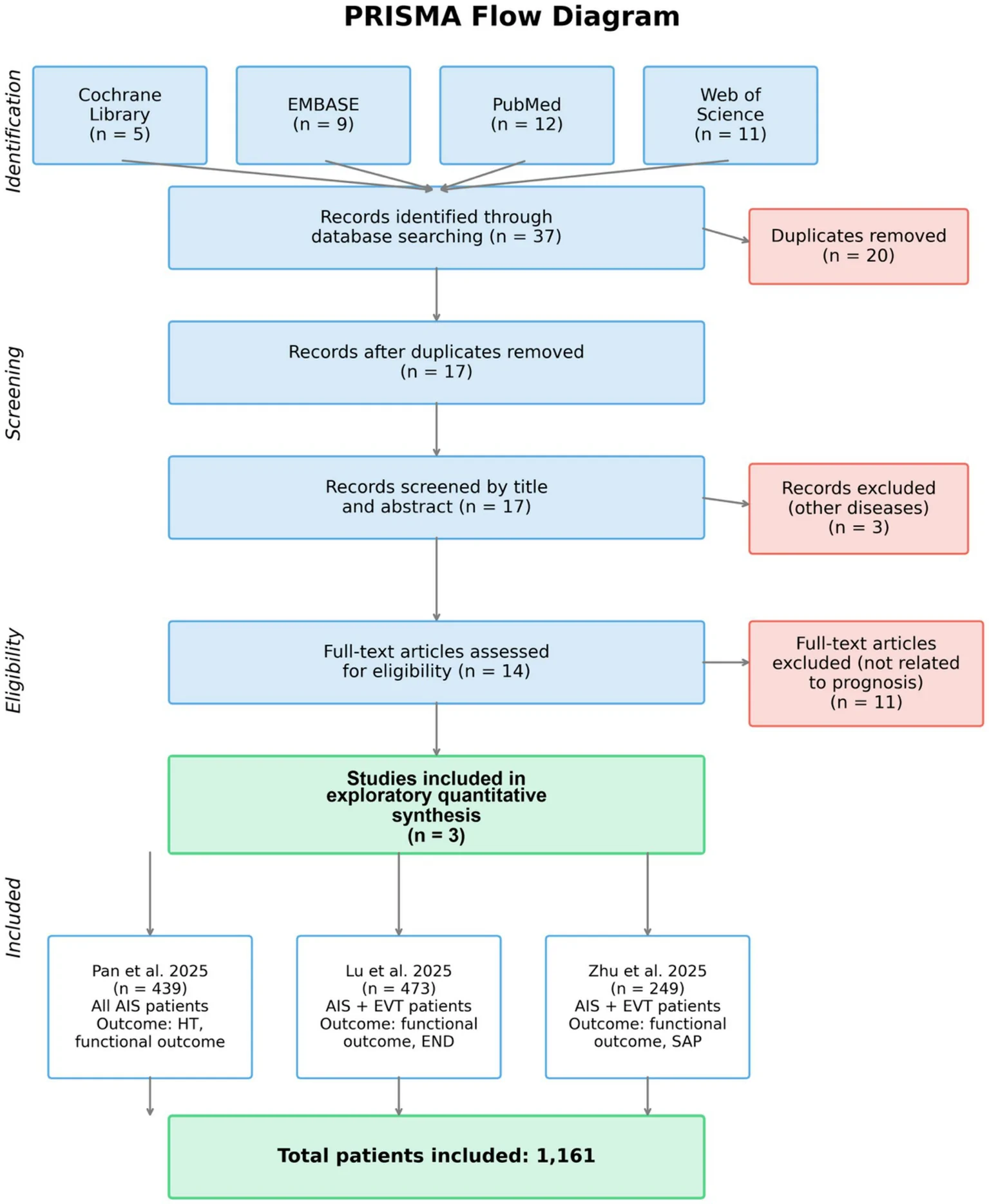
PRISMA flow diagram illustrating the study identification and selection process.

The initial database search yielded a total of 37 records: Cochrane Library (*n* = 5), EMBASE (*n* = 9), PubMed (*n* = 12), and Web of Science (*n* = 11). After removing 20 duplicate records, 17 articles remained for title and abstract screening. During screening, 3 records were excluded because they focused on other diseases unrelated to ischemic stroke. The remaining 14 articles underwent full-text assessment for eligibility, of which 11 were excluded as they did not evaluate the association between the CALLY index and stroke prognosis. Ultimately, 3 studies meeting all criteria were included in the exploratory quantitative synthesis (21, 25, 26). The PRISMA flow diagram detailing the study selection process is presented in Figure 1. An updated search in March 2026 identified no additional eligible studies.

### 3.2 Characteristics of included studies

The characteristics of the three included studies are summarized in Table 1. All three studies were retrospective observational studies conducted in China and published in 2025 (21, 25, 26). The total sample size was 1,161 patients with AIS. Pan et al. (21) enrolled 439 AIS patients admitted to Xiangya Hospital (December 2020–June 2023) and evaluated the CALLY index in relation to hemorrhagic transformation and 3-month functional outcome (mRS ≥ 2). Lu et al. (27) conducted a multicenter study involving 473 AIS patients treated with endovascular thrombectomy (EVT) at two centers (June 2021–September 2024), assessing 90-day functional outcome (mRS > 2) and early neurological deterioration. Zhu et al. (25) retrospectively reviewed 249 AIS patients who underwent EVT at Nanfang Hospital (January 2019–December 2023), evaluating the CALLY index for two primary outcomes: poor functional outcome (mRS ≥ 3) and stroke-associated pneumonia (SAP). All studies calculated the CALLY index using the same formula: albumin × lymphocyte count/(CRP × 10), and employed multivariable logistic regression analysis adjusting for relevant confounders. Blood sampling was performed within 24 h of admission [Pan et al. (21)]; prior to EVT in the emergency department [Lu et al. (27)]; within 24 h of admission [Zhu et al. (25)].

**Table 1:** Characteristics of studies included in the systematic review and adjusted ORs for the CALLY index (per unit increase) and poor functional outcome.

Study	Year	Design	*N*	Population	Outcome	OR (95% CI)	NOS
Pan et al. (21)	2025	Retro. Single-center	439	AIS (all treatments)	mRS ≥ 2 at 3 months	0.69 (0.56–0.86)	8/9
Lu et al. (27)	2025	Retro. Multi-center	473	AIS + EVT	mRS > 2 at 3 months	0.80 (0.70–0.91)	9/9
Zhu et al. (25)	2025	Retro. Single-center	249	AIS + EVT	mRS ≥ 3 at 3 months	0.76 (0.62–0.93)	9/9

AIS, acute ischemic stroke; CI, confidence interval; EVT, endovascular thrombectomy; mRS, modified Rankin Scale; NOS, Newcastle-Ottawa Scale; OR, odds ratio; Retro., retrospective.

### 3.3 Baseline characteristics of study populations

Table 2 presents the baseline demographic and clinical characteristics of patients enrolled in the included studies. The median age ranged from 62 to 71 years across studies, with a predominantly male population (63.8–75.5%). The prevalence of hypertension ranged from 64.3 to 72.7%, diabetes mellitus from 27.7 to 32.8%, and atrial fibrillation from 18.9 to 27.3%. Median baseline NIHSS scores varied from 4 to 13, reflecting differences in stroke severity across the study populations. Pan et al. (21) included a general AIS population, while Lu et al. (27) and Zhu et al. (25) specifically enrolled patients who underwent EVT for large vessel occlusion. Age is reported as median (IQR) or mean ± SD as originally presented in each study.

**Table 2:** Baseline characteristics of patients in the included studies.

Characteristic	Pan et al. (21) (*n* = 439)	Lu et al. (27) (*n* = 473)	Zhu et al. (25) (*n* = 249)
Age (years), median	64 (55–71)^a^	71 (64–79)^a^	62.1 ± 12.6^b^
Male, *n* (%)	311 (70.8%)	302 (63.8%)	188 (75.5%)
Hypertension, *n* (%)	319 (72.7%)	338 (71.5%)	160 (64.3%)
Diabetes mellitus, *n* (%)	144 (32.8%)	131 (27.7%)	79 (31.7%)
Atrial fibrillation, *n* (%)	93 (21.2%)	129 (27.3%)	47 (18.9%)
NIHSS, median (IQR)	4 (2–8)	13 (10–17)	11 (6–16)
IVT, *n* (%)	67 (15.3%)	179 (37.8%)	40 (16.1%)
CRP, median (IQR), mg/L	4.63 (2.57–11.40)	8.2 (3.0–27.0)	NR
Albumin, median, g/L	39.1 (36.5–41.3)	38.9 (35.5–41.9)	NR
Lymphocyte count, median (IQR), ×10^9^/L	1.5 (1.1–1.9)	1.0 (0.7–1.5)	1.33 (0.83–1.72)
CALLY index, median	1.17 (0.48–2.35)^a^	NR	0.80 (0.20–1.93)^a^
Poor outcome, *n* (%)	182/369 (49.3%)	214 (45.2%)	123 (49.4%)

CALLY, C-reactive protein-albumin-lymphocyte; CRP, C-reactive protein; IQR, interquartile range; IVT, intravenous thrombolysis; LYM, lymphocyte; NIHSS, National Institutes of Health Stroke Scale; NR, not reported; ^a^Median (IQR); ^b^Mean ± SD.

### 3.4 Exploratory quantitative synthesis of CALLY index and poor functional outcome

**Figure 2:**
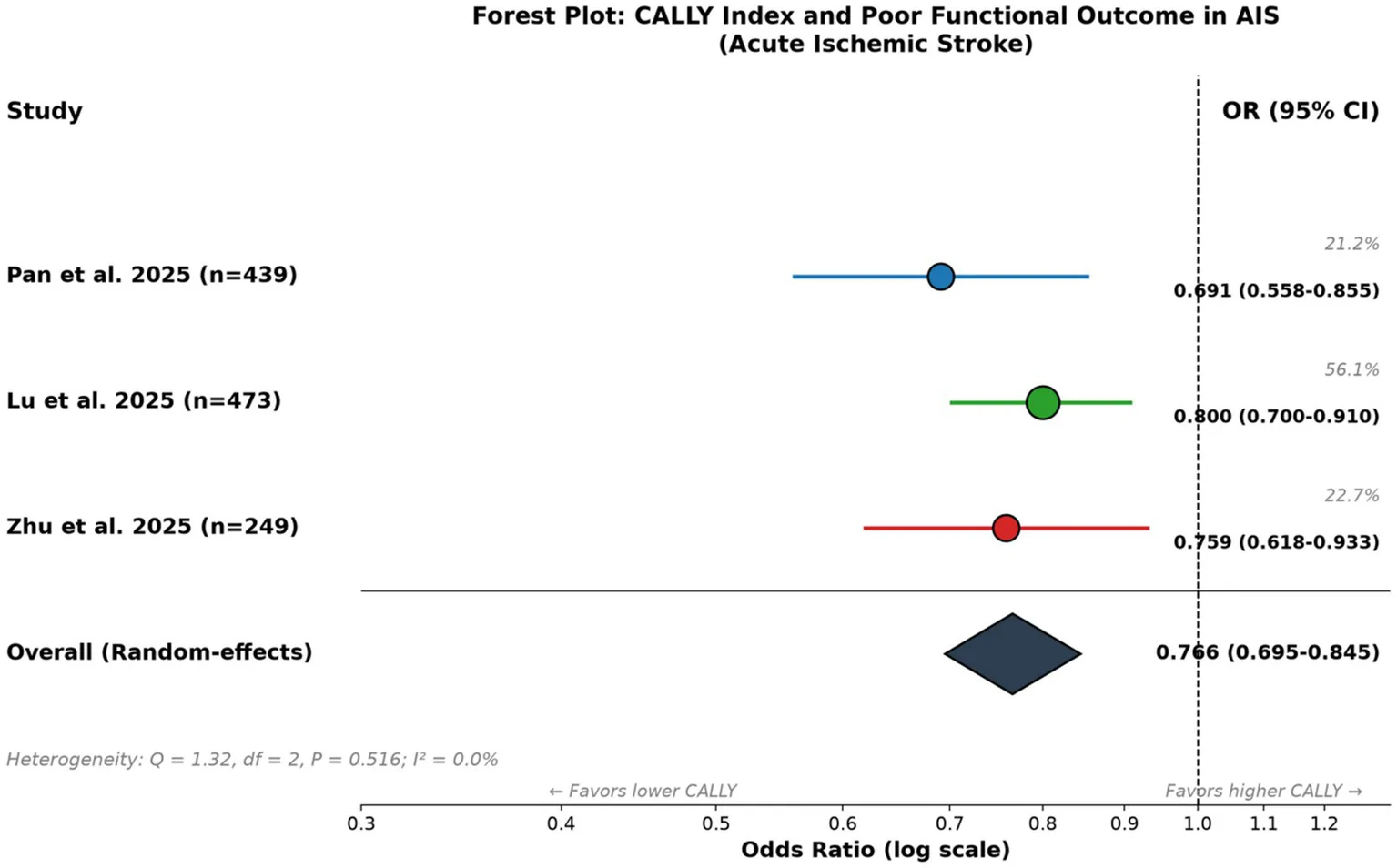
Forest plot of the association between CALLY index and poor functional outcome in AIS patients. The pooled OR was calculated using a random-effects model. The diamond represents the pooled estimate.

All three studies reported adjusted ORs for the association between the CALLY index (as a continuous variable) and poor functional outcome. The exploratory pooled analysis using a random-effects model demonstrated that a higher CALLY index was significantly associated with a reduced risk of poor functional outcome (pooled OR = 0.766, 95% CI: 0.695–0.845, *p* < 0.001) (Figure 2). No significant heterogeneity was detected across studies (*Q* = 1.33, d*f* = 2, *p* = 0.516; *I*^2^ = 0.0%), indicating consistent findings across the included studies. The weight distribution was as follows: Lu et al. (27) contributed 56.1%, Zhu et al. (25) 22.7%, and Pan et al. (21) 21.2%. However, the absence of statistical heterogeneity should be interpreted cautiously given the limited number of included studies and the consequently low power of Cochran’s Q test to detect between-study variance. Given that this pooled estimate is derived from only three retrospective studies, it should be considered exploratory and hypothesis-generating rather than confirmatory.

It should be noted that the included studies represent distinctly different clinical populations, which limits direct comparability. Pan et al. (21) studied a general AIS population with a wide range of stroke severity (median NIHSS = 4), including patients who received various treatment modalities (medical management, IV thrombolysis, or no acute intervention). In contrast, Lu et al. (27) and Zhu et al. (25) specifically enrolled EVT-treated patients with large vessel occlusion, who typically have more severe strokes (median NIHSS = 13 and 11, respectively) and distinct pathophysiological profiles. EVT-treated patients face unique complications such as reperfusion injury, hemorrhagic transformation, and ineffective recanalization, which may differentially influence the prognostic value of the CALLY index. Furthermore, the EVT populations were younger (median age 62–71 vs. general AIS) and had higher rates of IV thrombolysis. These population differences should be considered when interpreting the pooled estimate, and subgroup analysis stratified by treatment modality would be informative but is not possible with only three studies. Additionally, outcome definitions varied across studies (mRS ≥ 2 vs. mRS > 2 vs. mRS ≥ 3). Despite these sources of clinical heterogeneity, the direction and magnitude of the association remained consistent, which is encouraging but requires confirmation in larger, more homogeneous datasets.

### 3.5 Sensitivity analysis

Leave-one-out sensitivity analysis was performed by sequentially excluding each study and recalculating the pooled effect estimate. The results are summarized in Table 3. The pooled OR remained statistically significant and directionally consistent in all three scenarios: excluding Pan et al. (21) (pooled OR = 0.788, 95% CI: 0.706–0.880, *I*^2^ = 0.0%), excluding Lu et al. (27) (pooled OR = 0.726, 95% CI: 0.627–0.841, *I*^2^ = 0.0%), and excluding Zhu et al. (25) (pooled OR = 0.769, 95% CI: 0.687–0.859, *I*^2^ = 0.0%). Notably, the effect estimate was most pronounced (lowest OR) when Lu et al. (27) (the largest study contributing 56.1% of the weight) was excluded, reflecting its dominant but not driving contribution to the pooled estimate. The consistency of results across all leave-one-out analyses supports the robustness of the overall finding.

**Table 3:** Leave-one-out sensitivity analysis for the association between CALLY index and poor functional outcome.

Analysis	Pooled OR (95% CI)	*I*^2^ (%)	*p*-value (heterogeneity)
Overall (all 3 studies)	0.766 (0.695–0.845)	0.0	0.516
Excluding Pan et al. (21)	0.788 (0.706–0.880)	0.0	—
Excluding Lu et al. (27)	0.726 (0.627–0.841)	0.0	—
Excluding Zhu et al. (25)	0.769 (0.687–0.859)	0.0	—

CI, confidence interval; OR, odds ratio.

Zhu et al. (25) additionally evaluated the association between the CALLY index and stroke-associated pneumonia (SAP) in EVT-treated AIS patients. After multivariable adjustment, the CALLY index was significantly associated with SAP (OR = 0.715, 95% CI: 0.555–0.921, *p* = 0.009), suggesting that a lower CALLY index independently predicted increased risk of SAP. When analyzed as a categorical variable, patients with a CALLY index <0.83 had a significantly higher risk of SAP compared to those with a CALLY index ≥ 0.83 (OR = 2.123, 95% CI: 1.011–4.462, *p* = 0.047). However, the CALLY index was not significantly associated with malignant cerebral edema or early neurological deterioration after multivariable adjustment in the same study.

### 3.6 Stroke-associated pneumonia

Because these findings regarding SAP are derived from a single observational study, they should be considered exploratory and require external validation. No pooled analysis was possible for SAP, malignant cerebral edema, or early neurological deterioration due to insufficient studies reporting these outcomes.

### 3.7 Quality assessment

**Figure 3:**
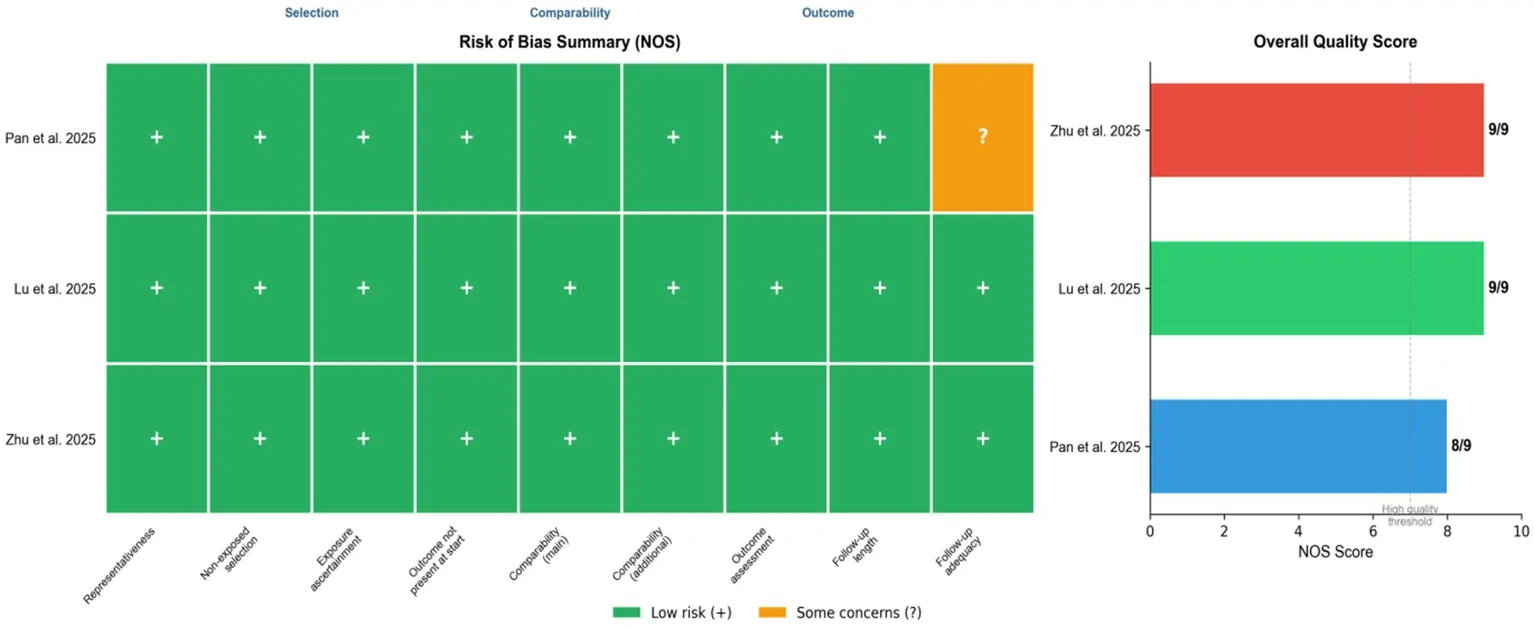
Risk of bias assessment using the Newcastle-Ottawa Scale (NOS). Left panel: Individual domain assessment for each study. Right panel: Overall NOS scores. Green (+) indicates the criterion was met; yellow (?) indicates some concerns.

The quality assessment results using the Newcastle-Ottawa Scale are presented in Figure 3. All three studies were rated as high quality, with NOS scores of 8 [Pan et al. (21)] and 9 [Lu et al. (27) and Zhu et al. (25)] out of a maximum of 9 stars. In the selection domain, all studies received full marks for adequate case definition, representativeness, exposure ascertainment, and demonstration that the outcome was not present at the start of the study. All studies scored the maximum of 2 stars for comparability, as they employed multivariable logistic regression adjusting for key confounders including age, sex, NIHSS score, and comorbidities. For the outcome domain, all studies used validated outcome measures (mRS) assessed by trained clinicians, with adequate follow-up periods (3 months). Pan et al. (21) lost one point in the follow-up adequacy category due to a 16% loss to follow-up rate (70/439 patients).

### 3.8 Publication bias

Publication bias was not assessed because the number of included studies was insufficient. Therefore, the possibility of publication bias cannot be excluded. While NOS scores indicate higher methodological quality, they do not eliminate the inherent limitations of retrospective observational designs, including potential residual confounding and unmeasured confounders.

## 4 Discussion

To our knowledge, this is the first systematic review with exploratory quantitative synthesis evaluating the CALLY index for functional outcomes in patients with acute ischemic stroke (AIS). Our analysis of three studies involving 1,161 patients showed that a higher CALLY index was significantly associated with a reduced risk of poor functional outcome (pooled OR = 0.766, 95% CI: 0.695–0.845), with no statistically detectable heterogeneity (*I*^2^ = 0.0%). These findings provide encouraging preliminary evidence suggesting that the CALLY index may serve as a practical and accessible prognostic biomarker in AIS. However, the pooled estimate should be interpreted as hypothesis-generating given the limited number of included studies.

The consistency of findings across the included studies suggests potential reproducibility, although external validation in diverse populations remains necessary. However, it should be emphasized that all studies originated from China, two studies included EVT-only populations, and no external validation cohorts exist. Therefore, generalizability to non-Chinese populations or non-EVT-treated patients requires further investigation.

The existing literature strongly supports the biological plausibility of the association between the CALLY index and AIS prognosis (28). The CALLY index integrates three key pathophysiological dimensions related to stroke outcomes. First, C-reactive protein (CRP), as a major acute-phase reactant, reflects the degree of systemic inflammation (29, 30). Neuroinflammation plays a central role in blood–brain barrier disruption, infarct expansion, and secondary brain injury after AIS (31, 32). Elevated CRP levels are consistently associated with poor functional outcomes and increased complications after pharmacological and mechanical reperfusion therapies (33, 34). Second, albumin is a marker of nutritional status and possesses anti-inflammatory, antioxidant, and antiplatelet properties (27, 35). Low serum albumin levels are associated with larger infarct volumes, elevated markers of blood–brain barrier disruption, and worse neurological outcomes (36, 37). Third, lymphocyte count reflects immune competence. Stroke-induced immunosuppression, characterized by lymphocytopenia, increases susceptibility to infection and impairs neurological recovery. By integrating these three dimensions, the CALLY index provides a more comprehensive assessment of a patient’s overall pathophysiological state than any single biomarker. The scientific significance of this association lies in its ability to transcend the limitations of single biomarkers, pointing toward an integrated perspective for post-stroke outcome assessment. The CALLY index integrates albumin (nutrition and liver function), lymphocytes (immune status), and C-reactive protein (systemic inflammatory intensity) into a single metric, potentially reflecting more holistically the body’s overall capacity to maintain homeostasis during the acute phase of stroke (28, 38). Therefore, the state represented by a higher CALLY index—characterized by “low inflammation, high nutrition, and strong immunity”—may collectively promote better functional recovery by mitigating neuroinflammatory damage, reducing the risk of infectious complications (such as stroke-associated pneumonia, SAP), and supporting neural repair processes.

Regarding the comparative value of the CALLY index relative to established prognostic markers, all three included studies reported that the CALLY index demonstrated moderate discriminative ability (AUC ranging from 0.63 to 0.76). Lu et al. (27) reported that the CALLY index outperformed the NLR, SII, and PLR in predicting poor functional outcome. Zhu et al. (25) demonstrated that the CALLY index had superior discriminative power compared to PNI and CONUT scores, with significant improvements in both NRI and IDI. Pan et al. (21) showed superiority over six established prediction scores for hemorrhagic transformation. These findings suggest that the CALLY index may provide incremental prognostic information beyond individual inflammatory or nutritional markers, although direct head-to-head comparisons with established composite scores (such as NIHSS-based models) in independent validation cohorts are needed to establish its added clinical value.

Regarding secondary outcomes, Zhu et al. (25) reported that a lower CALLY index was independently associated with stroke-associated pneumonia (SAP), which is one of the leading causes of morbidity and mortality in AIS. This finding aligns with the established understanding that immunosuppression and malnutrition synergistically increase susceptibility to post-stroke infection. However, because this observation was derived from a single study, further validation is required before clinical application. No pooled analysis was possible for SAP, and this finding should be considered hypothesis-generating. Traditionally, the occurrence of post-stroke pneumonia is closely linked to aspiration and immunosuppression (stroke-induced immunodepression syndrome) (39). The low lymphocyte count in the CALLY index directly reflects the immunosuppressed state, while low albumin and high CRP are associated with malnutrition and systemic inflammatory response, both of which are key factors for infection susceptibility (28, 40). Therefore, the CALLY index may serve as an early, easily accessible indicator for identifying AIS patients at high risk for SAP due to systemic inflammation-nutrition-immunity imbalance.

It is noteworthy that this analysis also found no significant association between the CALLY index and malignant brain edema. The currently available evidence does not demonstrate an association between the CALLY index and malignant cerebral edema, although this conclusion is based on a single study and additional investigations are required. If confirmed, this finding might suggest that the pathophysiological processes reflected by the CALLY index are more closely associated with systemic complications and long-term functional recovery than with acute local brain injury pathways, such as blood–brain barrier disruption and local cytotoxic edema. These findings may help clinicians more precisely define the clinical contexts in which this biomarker is most informative.

From a clinical perspective, the CALLY index offers several practical advantages: it relies solely on routine laboratory parameters (CRP, albumin, and lymphocyte count) readily available in Emergency Departments worldwide, requires no additional imaging or complex scoring, and enables rapid bedside calculation. The state represented by a higher CALLY index—characterized by “low inflammation, high nutrition, and strong immunity”—may theoretically promote better functional recovery by mitigating neuroinflammatory damage, reducing infectious complications, and supporting neural repair (41–43). However, it must be emphasized that the included studies did not demonstrate that CALLY-guided interventions improve outcomes. The potential clinical utility of the CALLY index for risk stratification, monitoring intensity decisions, or infection prevention strategies remains speculative and should be investigated in future interventional studies.

The CALLY index has also shown associations with disease risk in non-stroke populations, including hypertensive patients (38, 40) and those with chronic conditions such as diabetes and asthma, supporting its potential as a broad indicator of systemic health status. However, these observations do not directly support its use in guiding stroke treatment decisions, which would require dedicated prospective interventional studies.

The prognostic value of the CALLY index has been widely validated in oncology, where it independently predicts survival and surgical outcomes in various cancers (11, 44, 45), and a large-scale meta-analysis confirmed its association with all-cause mortality and disease progression (40, 46, 47). This cross-disease evidence supports the concept that systemic inflammation-nutrition-immunity balance is a key determinant of clinical outcomes under major physiological stress, whether from cancer or stroke. However, extrapolation from oncological to stroke populations should be done cautiously, as the pathophysiological contexts differ fundamentally.

Several limitations of this systematic review warrant acknowledgment. First, the number of included studies is limited to three, which substantially restricts the statistical power of the analysis and precludes robust assessment of publication bias through formal statistical tests. With only three studies, chance findings remain possible, and robustness cannot be confidently established. Second, all studies were conducted in Chinese populations, which may limit the generalizability of the findings to other ethnic and geographic regions. Third, the included studies were all retrospective in design, carrying inherent risks of selection bias, information bias, and residual confounding. Although multivariable-adjusted ORs were used, observational studies cannot eliminate unmeasured or residual confounding, and treatment-selection bias may influence the observed associations. Fourth, the definitions of poor functional outcome differed across studies: Pan et al. (21) used mRS ≥ 2 (includes patients with slight disability who can manage their own affairs without assistance), Lu et al. (27) used mRS > 2 (i.e., mRS ≥ 3, moderate-to-severe disability), and Zhu et al. (25) used mRS ≥ 3. These cut-offs are clinically distinct: mRS ≥ 2 captures a broader population including patients with minimal residual symptoms, whereas mRS ≥ 3 identifies only those with functionally significant disability requiring assistance. This variation in outcome thresholds may affect the sensitivity and specificity of the CALLY index as a predictor, as a biomarker may perform differently when predicting mild versus severe disability. Although no significant statistical heterogeneity was observed (*I*^2^ = 0.0%), the low power of the *Q* test with only three studies means that true clinical heterogeneity cannot be excluded. Fifth, only baseline CALLY index values were analyzed; the potential prognostic value of dynamic changes in the CALLY index during hospitalization remains to be explored. Sixth, the pooled estimate was dominated by a single study [Lu et al. (27), contributing 56.1% of the pooled weight], which may limit the representativeness of the pooled estimate. Finally, the total sample size is relatively small (*n* = 1,161), and the included populations are not fully comparable: one study enrolled general AIS patients while two studies included only EVT-treated patients with large vessel occlusion, limiting the generalizability of the pooled estimate. Additionally, the variation in mRS thresholds across studies (mRS ≥ 2, mRS > 2, and mRS ≥ 3) represents clinically different outcome definitions that may affect the interpretation of the CALLY index’s predictive performance.

## 5 Conclusion

This systematic review with exploratory quantitative synthesis provides preliminary pooled evidence suggesting that the CALLY index may be a potential predictor of poor functional outcome in AIS patients. A higher CALLY index was associated with a 23.4% reduction in the odds of poor functional outcome (pooled OR = 0.766, 95% CI: 0.695–0.845). All included studies demonstrated high methodological quality, and no statistically significant heterogeneity was observed, although the limited number of studies restricts interpretation. The CALLY index, derived from routinely available laboratory parameters, offers a potentially practical tool for early prognostic assessment in AIS patients. However, given that the current evidence is based on only three retrospective studies from China, these findings should be considered hypothesis-generating rather than confirmatory. Large-scale, multicenter, prospective studies across diverse populations are needed to validate these preliminary findings before any clinical implementation.

## Data Availability

The raw data supporting the conclusions of this article will be made available by the authors, without undue reservation.
